# The Effect of a Subsequent Dose of Dexmedetomidine or Other Sedatives following an Initial Dose of Dexmedetomidine on Sedation and Quality of Recovery in Cats: Part I

**DOI:** 10.3390/vetsci11050186

**Published:** 2024-04-25

**Authors:** Chrysoula Margeti, Georgios Kazakos, Vassilis Skampardonis, Apostolos D. Galatos, Theodora Zacharopoulou, Vassiliki Tsioli, Epameinondas Loukopoulos, Panagiota Tyrnenopoulou, Vasileios G. Papatsiros, Eugenia Flouraki

**Affiliations:** 1 Clinic of Surgery, Faculty of Veterinary Medicine, School of Health Sciences, University of Thessaly, Trikalon 224, 43100 Karditsa, Greece; margeti@uth.gr (C.M.); agalatos@vet.uth.gr (A.D.G.); zacharop@uth.gr (T.Z.); vtsioli@uth.gr (V.T.); nlouk09@gmail.com (E.L.); ptyrnenop@uth.gr (P.T.); 2 Companion Animal Clinic, School of Veterinary Medicine, Aristotle University of Thessaloniki, 54627 Thessaloniki, Greece; gkdvm@vet.auth.gr; 3 Department of Epidemiology, Biostatistics and Animal Health Economics, Faculty of Veterinary Medicine, School of Health Sciences, University of Thessaly, Trikalon 224, 43100 Karditsa, Greece; bskamp@uth.gr; 4 Clinic of Medicine, Faculty of Veterinary Medicine, School of Health Sciences, University of Thessaly, Trikalon 224, 43100 Karditsa, Greece

**Keywords:** cat, consecutive doses, dexmedetomidine, drug combination, ketamine, midazolam, opioids, recovery, sedation

## Abstract

**Simple Summary:**

Dexmedetomidine is a frequently used sedative; however, in some cases, its administration may prove insufficient; therefore, an additional dose or another drug is required to achieve the desired sedative outcome. The present study investigated the sedative effects of commonly used anaesthetics when administered after an inadequate initial dose of dexmedetomidine. Six healthy adult cats were included in the study, and each cat participated seven times. The initial dose of dexmedetomidine was followed by the administration of a second dose of dexmedetomidine, butorphanol, buprenorphine, tramadol, ketamine, midazolam, or saline NS 0.9%. Additionally, atipamezole was administered to all animals to elicit recovery. To assess the sedative effect of each treatment, a sedation scale was used, and recovery quality was evaluated using two recovery scales. The results suggested that the two consecutive doses of dexmedetomidine produced deeper sedation compared to the administration of a single dose of dexmedetomidine. Sedation levels were also enhanced by the administration of dexmedetomidine, butorphanol, or ketamine following the administration of an initial dose of dexmedetomidine, and the recovery quality was good. On the contrary, administration of midazolam following dexmedetomidine administration resulted in inferior sedation and recovery quality.

**Abstract:**

Dexmedetomidine is an a_2_-agonist commonly used in veterinary practice. Occasionally, the administered dose of dexmedetomidine may result in insufficient sedation, and an additional dose or drug may be required. The sedative effects of seven different drugs administered at subsequent time points after an initial, insufficient dose of dexmedetomidine were evaluated. Seven adult cats participated in this crossover, blind, randomised study. The groups consisted of two consecutive doses of dexmedetomidine (15 + 10 μg/kg) (DD) or a dose of dexmedetomidine (15 μg/kg) followed by either NS 0.9% (DC-control group), tramadol 2 mg/kg (DT), butorphanol 0.2 mg/kg (DBT), buprenorphine 20 μg/kg (DBP), ketamine 2 mg/kg (DK), or midazolam 0.1 mg/kg (DM). Sedation was evaluated using the Grint sedation scale. In all groups, atipamezole was administered at the end of the evaluation, and recovery was assessed using the Lozano and Sams recovery scales. The DC and DM groups exhibited minimal sedative effects. The maximum sedative effect was observed in the DD and DK groups, while sedation in the DD and DK groups was significantly higher compared to the DC group. Recovery in all groups was uneventful, except in the DM group, where it was prolonged and difficult, although no statistically significant difference was detected. Therefore, insufficient sedation with dexmedetomidine can be enhanced by a subsequent dose of dexmedetomidine, ketamine, or butorphanol, whereas the addition of midazolam reduces sedation and prolongs recovery.

## 1. Introduction

Alpha_2_-adrenergic receptor agonists (a_2_-agonists) are products of the prototype drug clonidine [1]. They are widely used in veterinary practice to elicit dose-dependent and consistent sedative, analgesic, and sympatholytic effects [1,2]. Dexmedetomidine is a highly selective a_2_-agonist that demonstrates cardiovascular and respiratory depression as well as metabolic dysregulation [2,3,4]. A fundamental attribute of a_2_-agonists lies in their susceptibility to antagonism by atipamezole or yohimbine [2,5].

A_2_-agonists manifest analgesic and sedative effects and are frequently combined with anaesthetic or analgesic drugs to increase their effects and reduce the required doses, thereby reducing possible adverse effects [2,6,7,8]. Butorphanol is an agonist of κ-opioid receptors and an antagonist of μ-opioid receptors [9,10], which provides mild visceral analgesia and sedation [11,12,13]. Buprenorphine, a partial agonist of the μ-opioid receptors, is very often used in cats for mild to moderate analgesia and sedation [8,14,15]. Furthermore, tramadol, a centrally acting synthetic opioid, provides analgesic and sedative effects through μ-opioid, serotonin, and adrenergic receptor interactions [16,17]. Ketamine is a widely used anaesthetic in cats, as it induces dissociative anaesthesia and analgesia without causing significant cardiovascular depression [18]. Finally, benzodiazepines can induce drug- and species-dependent sedation. Midazolam is a synthetic benzodiazepine with sedative, muscle-relaxing, and anticonvulsant effects and minimal effects on the cardiovascular system [19].

Repeated administration of sedative drugs can influence the degree and duration of sedation as well as the quality of recovery. Under certain circumstances, the administered dose of dexmedetomidine may fail to produce the anticipated sedative effects. The existing literature does not provide sufficient evidence on the impact of dexmedetomidine administered for a second time at a specific interval following an inadequate initial dose. The primary goal of our study was to assess the sedative effects, duration of action, and quality of recovery of two consecutive doses of dexmedetomidine in cats. Furthermore, the current literature provides a wealth of information on the combination of dexmedetomidine with various anaesthetic or analgesic drugs. The precise effects of administering these medications at specific intervals, particularly after a single dose of dexmedetomidine, remain unclear. Therefore, common anaesthetic drugs were administered after a single dose of dexmedetomidine, and the effects of different drug combinations were evaluated. The main hypotheses of our study are as follows:

- Following insufficient sedation resulting from a single administration of dexmedetomidine, the subsequent dose has the potential to enhance the quality, intensity, and duration of the sedative effect.
- Following insufficient sedation resulting from a single administration of dexmedetomidine, the administration of additional medications, such as common opioids or anaesthetic agents, enhances the sedative properties of dexmedetomidine.
- The administered drugs do not compromise the quality of recovery.

## 2. Materials and Methods

This study was a prospective, randomised order, blind, crossover, experimental study. It was approved by and followed the guidelines of the Animal Ethics Committee of Greece (licence number: 504050, date: 20 December 2021), which confirmed that it complied with the standards of national and EU legislation (Directive 2010/63/EU for animal experiments) regarding animal experimentation. Additionally, approval for this study was obtained from the Animal Ethics Committee (EDEXZO, number: 138, date: 24 March 2022) of the Department of Veterinary Medicine of the University of Thessaly (UTH). This study was a two-part series of articles. The second part of our article can be found under the title: “The effect of a subsequent dose of dexmedetomidine or other sedatives following an initial dose of dexmedetomidine on electrolytes, acid–base balance, creatinine, glucose, and cardiac troponin I in cats: Part II”.

Six healthy adult purpose-bred laboratory domestic shorthaired (DSH) cats were enrolled in the study: five males and one female, aged 3–4 years and weighing 2.5–4 kg. Throughout the study, the animals were housed in specially constructed cages inside a calm and quiet room, apart from other animals. The cats were accustomed to the room and the personnel. The researchers were experienced in cat handling, and meticulous techniques were used to minimise stress. The cats were classified as American Society of Anaesthesiologists (ASA) status 1. The physical status of the animals was thoroughly examined before commencing the experiments. Clinical examination included body weight assessment, thoracic auscultation, assessment of heart rate (HR) and respiratory rate (RR) and rhythm via auscultation, temperature measurement, capillary refill time measurement, and pulse character estimation by palpation. A total blood count was performed, including measurements of red blood cells, haemoglobin (Hb), haematocrit (HCT), white blood cell count, and type and platelet count. Serum biochemical profiles were also obtained, including measurements of creatinine (CREA), total proteins, albumin, alkaline phosphatase, alanine aminotransferase, glucose (Glu), urea, potassium (K^+^), and sodium (Na^+^). Animals with abnormal findings were not included in the study. Other exclusion criteria included aggressive behaviour, obesity, history of seizures, cardiac disease, and any impairment in physical condition.

Randomisation was performed using the GraphPad by DotMatics (Boston, MA, USA) online randomiser (https://www.graphpad.com/quickcalcs/randomize1/ accessed on 25 March 2022). Three examiners participated in the study to ensure the objectivity of the measurements. The main examiner (C.M.) was blinded to the experimental groups and performed all assessments and administrations. The second examiner (T.Z.), who was blinded to the study, assisted with animal restraint, whereas the third participant (E.L.) was responsible for all medication preparation and administration and had no other involvement in the assessments.

Each animal participated in the study seven times, with a washout period of seven days [7]. The study included seven experimental groups, in which different drugs were administered at different time points. In all animals, the first injection consisted of 15 μg/kg dexmedetomidine (Dexdomitor, Orion Pharma, Espoo, Finland). The second administration was performed at the following time point with one of the additional drugs. The second administration consisted of either dexmedetomidine at 10 μg/kg (DD group), NS 0.9% 0.1 mL (DC or control group), butorphanol (Dolorex, MSD, Haarlem, The Netherlands) at 0.2 mg/kg (DBT group), buprenorphine (Bupaq, Neocell, Athens, Greece) at 20 μg/kg (DBP group), tramadol (Tramal, Vianex, Athens, Greece) at 2 mg/kg (DT group), ketamine (Ketaset, Zoetis, Athens, Greece) at 2 mg/kg (DK group), or midazolam (Dormicum, Cheplapharm, Greifswald, Germany) at 0.1 mg/kg (DM group).

All injections were administered intramuscularly (IM) to the quadriceps muscle to ensure stability of absorption [20]. 

The sedation score was assessed at the time of baseline measurements and repeated throughout the experiment using the composite descriptive sedation score by Grint et al. (2009) [21], adapted from Young et al. (1990) [22] and Kuusela et al. (2000) [23], with reported use in cats by Deutsch et al. (2017) [24]. The scores on this scale range from 0 to 21, with zero indicating no sedation and 21 indicating deep sedation. A score of 0–3 indicates no sedation; a score of 4–12 indicates moderate sedation; and a score of 13–21 indicates deep sedation. Seven attributes were examined, namely spontaneous posture, palpebral reflex, eye position, jaw and tongue relaxation, response to noise, resistance when laid laterally, and general appearance. The initial dose of dexmedetomidine was intended to produce mild sedation (>4 score on the Grint scale). The additional dose was intended to increase the sedative level to moderate (>8 score in the Grint scale). The intended sedation would be adequate for mild procedures, such as a clinical examination or blood collection, with minimal handling of the animal.

The recovery quality was assessed using the following scales:

1. The Simple Descriptive Scale (SDS), developed by Lozano et al. (2009) [25], has not been previously used in cats. The scores on this scale ranged from 1 to 5, with 1 indicating smooth recovery and 5 indicating extremely violent recovery.
2. Ataxia, induction, and recovery quality scores were reported by Sams et al. (2008) [26], which were reportedly used in cats by Kim et al. (2015) [27]. The scores on this scale vary from 0 to 3, with 0 indicating perfect recovery and 3 indicating rough recovery.

Four time points were established (T0, T1, T2, and T3) (Table 1). T0 is the time point of the baseline measurements and the first drug administration. Following the first administration, the sedation score was evaluated every 5 min. Next, the time points T1 and T2 were established using the Grint scale. Time point T1 was the first moment in time when two consecutive Grint scale scores had the same value (other than zero). Thus, T1 was defined as the time of maximum sedation for the first drug. At T1, the measurements were repeated, and the second drug was administered. The next time point, T2, was established in the same manner as the point in time when two consecutive scores on the Grint scale had the same value. Therefore, T2 was defined as the point of maximum sedation for drug combinations. If the Grint score decreased in consecutive measurements, the last measurement was defined as time point T2. At T2, the measurements were repeated and atipamezole (Antisedan, Zoetis, Athens, Greece) was administered, except for the DK group, where atipamezole was administered at a following time point that was defined as T2_A_. Following the administration of atipamezole, recovery quality was assessed every five minutes using the two recovery scale scores. The moment that the cat was walking steadily, without signs of ataxia, was the full recovery time (T3), and the cat could return to its accommodation area. 

On the day of the experiment, food was withheld for ten hours, and water was abstained for one hour before its initiation [28]. At time point T0, a clinical examination was performed, the sedation score was evaluated, and the initial dose of dexmedetomidine was administered. 

At T1, the second drug was administered, as determined by animal grouping. Thus, at T1, appropriate doses of dexmedetomidine, saline (NS 0.9%), butorphanol, buprenorphine, tramadol, ketamine, or midazolam were administered.

At time point T2, the a_2_ antagonist, atipamezole, was administered IM at the recommended dose. In the DC, DT, DBT, DBP, DK, and DM groups, atipamezole was administered at a dose of 75 μg/kg and in the DD group at a dose of 125 μg/kg. In the DK group, the administration of atipamezole was delayed by 30 min following ketamine administration to minimise the risk of adverse effects [29]. Therefore, in the DK group, a specific time point was established (T2_A_) when atipamezole was administered. Moreover, all quality of recovery assessments in the DK group commenced 5 min after T2_A_.

### Statistical Analysis

The required sample’s size calculation was performed using G*Power (version 3.1.9.4) software, following an a priori type of power analysis, conditional on prespecified levels of significance, power, and effect size. The necessary pre-estimations were based on assumptions deduced from a pilot study using clinical cases in cats referred to our clinic, aiming primarily at the investigation of the sedative effect, measured in the Grint scale, of the administration of anaesthetic substances, namely butorphanol, midazolam, dexmedetomidine, and ketamine, following an inadequate initial sedation with dexmedetomidine. These results suggested significant differences in the average sedation degree between the initial administration of dexmedetomidine and the subsequent complementary administrations. The corresponding effect size (Cohen’s f) was calculated using the maximum and minimum average differences of Grint scale scores between groups, accounting accordingly either for minimum, intermediate, or maximum variability in the means’ distribution [30]. The resulted effect sizes (f = 0.309, f = 0.325, and f = 0.437, respectively) were used in equally numbered power analyses, assuming a repeated measurement design with seven groups and setting the confidence interval at 95%, significance level at 5%, and power at 95%. The required total sample size on each occasion was 35, 28, and 21 individual subjects, achieving power of 98%, 96%, and 99%, respectively, for each assumed effect size. However, due to the adopted cross-over design of our study, the assignment of the sequence of the seven drug combinations to each of the six cats resulted in 42 observations, satisfying the above maximum requirements of the least required sample size.

One of the main concerns of our analyses was the expected dependence of observations from repeated measurements over time within the same animal and the potential within each different animal-specific drug combination (6 animal × 7 drug combinations = 42 animal–drug combinations), resulting in a three-level hierarchical data structure design. According to the results of Shapiro–Wilk tests for normal data and based on the shape of the constructed histograms, the measured parameters, although continuous, were not normally distributed. For the Grint scores, the Wilcoxon signed rank test was also performed, assessing the change in their median values between time points T0–T1, T1–T2, and T0–T2. 

For each of the non–normally distributed parameters, a quantile regression model was employed to account for the distributional limitations of the parameters’ divergence from normality, assessing the effect of different drug combinations over T0, T1, and T2. Specifically, the qreg2 command was used to estimate quantile regression, allowing for the adjustment of standard errors and t–statistics that are asymptotically valid under heteroskedasticity and intra–cluster correlation between measurements within the same animal and within the same animal treatment combination, using the cluster option [31]. The presence or absence of correlation in each potential source of intra–cluster correlation (animal and specific animal group level) was tested with the Parente–Santos Silva test [31]. The median values of the non–normally distributed parameters were the dependent variables, while drug combination and administration points (T0, T1, and T2) were the respective independent ones for each corresponding model.

Estimation of the mean time to “maximum sedation from first drug administration”, “maximum sedation after second drug administration”, “treatment effect”, and “full recovery” was performed with the employment of equally numbered multilevel mixed-effects parametric survival models, addressing the inherent correlation and dependence of these time-to-event data within the same animal, formerly accounted for by parametric frailty and shared frailty survival models [32]. Initially, we assessed the fit to our data of five different parametric distributions, namely the exponential, the Weibull, the Gompetz, the log-normal, and the log-logistic, for each failure time. The selection of the most appropriate distribution was based on the shape of the hazard curves and the smallest Akaike’s information criterion (AIC) [33,34]. The interpretation of the multilevel mixed-effects parametric survival models was based on the accelerated failure time (AFT) metric. Time ratios (TRs) were used to assess the effect of the applied drug combinations; in our case, TRs > 1 suggested that drug combinations prolonged the mean time to onset of each of the events described above [33,35]. Hazard functions were estimated to assess the drug combination-specific change in the risk of the first occurrence of the event of interest.

The Lozano and Sams recovery quality scales could be considered discrete, categorical variables with five and four levels, respectively. The effect of the applied drug combinations on these recovery quality scales was assessed with the use of two distinct, one for each recovery scale, multilevel mixed-effects ordinal logistic regression models. The Lozano and Sams recovery scores were the dependent variables, whereas a dummy variable coding for the applied drug combinations was the independent one. A random effect term at the animal level was incorporated to account for the dependence arising from the correlation of observations within the same cat.

## 3. Results

Six cats participated in the experiment, with a mean age of 3.33 ± 0.5 years and a mean body weight of 3.55 ± 0.7 kg. The mean dexmedetomidine dose administered at T0 was 51.5 ± 10.3 μg, and the experiment lasted a mean of 42.6 ± 5.5 min (T0–T3). The mean dosages of the additional drugs at T1 were dexmedetomidine 32.5 ± 7.5 μg, tramadol 6.9 ± 1.3 mg, butorphanol 0.63 ± 0.13 mg, buprenorphine 69.5 ± 12.53 μg, ketamine 6.8 ± 1.3 mg, or midazolam 0.34 ± 0.06 mg.

### 3.1. Quality and Duration of Sedation 

The mean durations for all time intervals of the experiment are presented in Table 2.

The mean duration of T0–T1 was significantly longer in the DD group in comparison to groups DT by 22.5% (95% CI: 9.3; 43.8, *p* = 0.002), DBT by 23.3% (95% CI: 5.9; 37.4, *p* = 0.011), and DBP by 20% (95% CI: 5.6; 32, *p* = 0.008). The maximum mean duration of T0–T2 was recorded in the DD and DBP groups, whereas the minimum mean duration of T0–T2 was recorded in the DM and DT groups (Table 2). The comparison of the mean duration of T1–T2 and T0–T2 between the seven groups is presented in Table 3.

Grint scale scores were zero at baseline in all groups. The median sedation scale scores at T1 did not differ significantly between the groups (all *p* ≥ 0.06). The minimum median sedation scores at time point T2 were observed in DC and DM groups, whereas the maximum median sedation scores at T2 were observed in DD and DK groups (Table 4).

**Figure 1:**
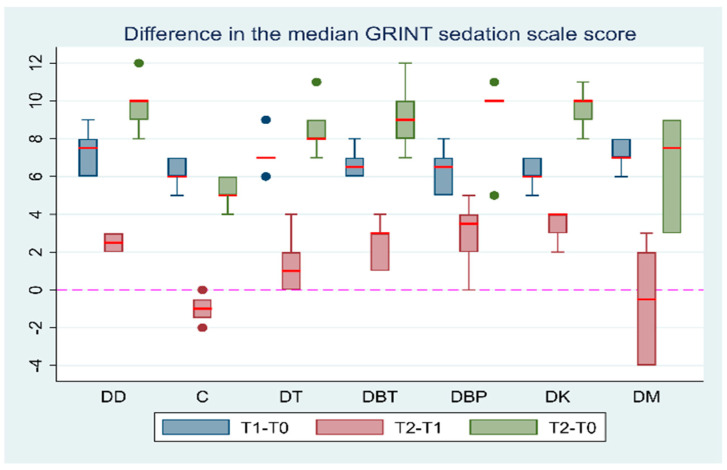
Grint scale score differences between T0 (baseline), T1 (maximum sedation with the first drug), and T2 (maximum sedation of the drug combination) for seven different groups (DD, DBP, DK, and DM) of six adult cats that received different drug combinations. Group DD: administration of two repeated doses of dexmedetomidine; DC: dexmedetomidine–NS 0.9% combination (control group); DT: dexmedetomidine–tramadol combination; DBT: dexmedetomidine–butorphanol combination; DBP: dexmedetomidine–buprenorphine combination; DK: dexmedetomidine–ketamine combination; DM: dexmedetomidine–midazolam combination. The red horizontal line in the middle of each box plot represents the median value. Dots represent the outliers of the box plots—observations far removed in value from the rest.

The median value of the Grint sedation scale was significantly higher at T1 than T0 (Coef.: 7, 95% CI: 6.58; 7.41, *p* < 0.001). The median values of the Grint sedation scale were significantly higher at T2 than at T0 in all groups (*p* = 0.0313). The median Grint sedation scale score was significantly higher at T2 than at T1 in the DD (*p* = 0.0313), DBT (*p* = 0.0313), and DK (*p* = 0.0313) groups. On the other hand, the median sedation score in the DC, DT, DBP, and DM groups (all *p* > 0.0625) had no statistically significant difference in T2 compared to T1. The comparisons of the median Grint scores between T1–T2 and T0–T2 can be found in the Supplementary Materials (Table S4). Comparisons of the median score differences in the sedation scales at T1–T2 and T0–T2 intervals between the groups are presented in Table 5. Additionally, the Grint sedation scale score differences between the time intervals are presented in Figure 1.

### 3.2. Quality and Duration of Recovery

Based on the shape of the hazard curves and the AIC value, the Weibull (AIC: 10.26684) distribution fitted our data best for time-to-full recovery (T3–T2) analysis. The maximum mean duration of T2–T3 was recorded in the DM (19.2 ± 1.9 min) and DK (17.3 ± 2.7 min) groups. The minimum mean duration of T2–T3 was recorded in the DBT group (11.3 min). The mean duration of T2–T3 is presented in Table 2. Comparisons of the mean duration of T2–T3 between the groups are presented in Table 6.

In the DK group, the time interval above was T2_A_–T3. In the DK group, the mean time from maximum sedation with the drug combination (T2) until the administration of atipamezole was 17.5 ± 2.7 min.

There were no significant differences in the median scores of the Lozano and Sams recovery quality scales between the groups at any time point during the recovery assessment (*p* > 0.99 and *p* > 0.058, respectively). However, the maximum median Lozano and Sams recovery scale scores were recorded in the DM group [Lozano median score: 2 (1–3) and Sams median score: 2 (1–2)]. Specifically, three out of six cats in the DM group recovered with a Lozano score of 2 and a Sams score of 2. One out of six cats in the same group recovered with a Lozano score of 3 and a Sams score of 2. All other cats in all groups recovered smoothly (Lozano score 1), without complications, and with minimal or no ataxia (Sams score 0–1).

## 4. Discussion

Dexmedetomidine is frequently used in clinical settings as a pre-anaesthetic agent or as a sedative. A_2_-agonists provide reliable sedation and anxiolysis through norepinephrine outflow reduction within the central nervous system (CNS), which decreases central sympathetic tone [36]. The use of dexmedetomidine has progressively increased in recent years, especially in combination with other anaesthetic drugs [8]. The most frequently recommended single dose of dexmedetomidine in cats is 40 μg/kg [3,37,38]. In a study by Granholm et al., administration of that dose resulted in moderate to intense sedation, which allowed various minor operations to be performed [3]. Accordingly, the analgesic effect of dexmedetomidine administered IM and the degree of sedation caused by different doses were found to be dose-dependent [8]. Doses of 2, 5, or 10 μg/kg failed to provide sufficient sedative or analgesic effects. Administration of 20 μg/kg dexmedetomidine resulted in adequate sedation in cats, and a dose of 40 μg/kg provided the most potent and prolonged sedation and analgesia [8]. In the clinical setting, adequate sedation is not always achieved by the administered dose of dexmedetomidine. Our study aimed to assess whether insufficient sedation with dexmedetomidine can be enhanced by a second dose of dexmedetomidine or by the addition of other sedatives or opioids. Therefore, we chose a dose of 15 μg/kg to achieve a sedative effect between 10 μg/kg (reported as insufficient) and 20 μg/kg (reported as adequate). In group DD, the total dose of dexmedetomidine was 25 μg/kg, which did not exceed the recommended dose and resulted in an adequate level of sedation according to the literature.

Dexmedetomidine is usually administered in combination with opioids or other anaesthetic drugs to enhance sedation and reduce the required doses. The opioids selected for this study were butorphanol, buprenorphine, and tramadol. These opioids are easily accessible to the clinician in comparison to other opioids, like pure μ-opioid agonists. Moreover, we avoided the use of pure μ-opioid agonists since there is a global tendency towards opioid-free anaesthesia in both human and veterinary medicine. Nevertheless, opioids are the foundation of acute pain management, and their use cannot be completely overthrown; thus, opioid-sparing anaesthesia is preferred [39]. Ketamine and midazolam are also frequently administered in combination with dexmedetomidine in clinical practice. The combination of ketamine with a_2_-agonists has been reported in various studies in cats [40,41,42,43,44] and dogs [45,46,47,48,49]. This combination causes predictable and adequate sedation, analgesia, and a good quality of recovery [40,44,50]. Additionally, midazolam is often administered in combination with a_2_-agonists [51,52] and/or ketamine [40,43,53] to improve the sedative effect of these drugs.

### 4.1. Quality and Duration of Sedation

In our study, the maximum level of sedation (T1) after the administration of 15 μg/kg dexmedetomidine was moderate (score range of 4–12) according to the Grint sedation scale [21]. These results are in accordance with various studies where the administration of 10–25 μg/kg dexmedetomidine induced moderate sedation [11,50,54]. The mean time to reach maximum sedation (T1) after dexmedetomidine administration (T0) was 17 ± 3.05 min. Similar results were observed in a study in cats in which the sedative effect of various doses of dexmedetomidine was evident within 5–13 min after IM administration and peak sedation was achieved within 20–30 min [3,11,50].

It has been reported that a higher dose of dexmedetomidine (30 μg/kg in comparison to 15 μg/kg) increased the duration of sedation but not the sedation level in cats [54]. Additionally, the same result was reported in dogs after administration of 10 and 20 μg/kg dexmedetomidine [23]. The findings of the present study differ from those of the aforementioned studies. In our study, according to the Grint sedation scale, the sedation level in the DD group was significantly increased by the second dose of dexmedetomidine between T1 and T2 (*p* = 0.0313). Furthermore, the median Grint sedation scale score was significantly higher (*p* = 0.006) in the DD group than in the DC group after the second administration (interval T1–T2). Therefore, more profound sedation was demonstrated by the administration of two consecutive doses of dexmedetomidine (15 + 10 μg/kg) in comparison with the control group (DC), with a statistically significant difference with the Grint sedation scale. In the DC group, the sedative effect of dexmedetomidine gradually decreased from T1 to T2, while the sedation level remained moderate.

The mean durations of T1–T2 and T0–T2 were significantly lower in the DM group than in the DC group (*p* = 0.001 and *p* = 0.016, respectively). Therefore, midazolam administration had a negative effect on the duration of dexmedetomidine sedation when administered consecutively. In the present study, the effect of midazolam was noticeable promptly, and time point T2 was reached within 8.33 ± 2.3 min. A study by Ilkiw et al. (1996) reported that the mean time to onset of action of midazolam after an IM administration of 0.5 mg/kg in cats was rapid (2.38 ± 1.74 min), and its effects lasted for 30–60 min [19]. The sedative effect of various doses (0.05–5 mg/kg) of midazolam alone was minimal, while ataxia, dysphoria, agitation, struggling, or behavioural modification were often documented [19,55]. These excitement-like symptoms could be interpreted as lower sedation levels when sedation scales are implemented. Similar results were observed in the present study. At time point T2, in the DM group, the sedation score was lower than that at T1, without a statistically significant difference. Likewise, it has been reported that IM administration of midazolam (0.5 or 0.4 mg/kg) alone or in combination with butorphanol did not cause apparent sedation; however, excitement-like behaviours were profound [40,52]. The same result was not observed after the administration of midazolam–butorphanol–dexmedetomidine, midazolam–butorphanol–ketamine [40], or midazolam–methadone–dexmedetomidine combinations [51]. It appears that sedative drugs, such as dexmedetomidine or ketamine, in high doses, combined with an opioid, could conceal the behavioral modification of midazolam. The same result was not apparent in the present study, in which a low dose of dexmedetomidine was combined with midazolam without the concurrent administration of an opioid.

In the present study, a significant increase in sedation was observed after the administration of ketamine, whereas maximum sedation was apparent within 12.5 ± 2.5 min. Likewise, various studies have reported that dexmedetomidine and ketamine have synergistic sedative effects [40,50,56]. The administration of dexmedetomidine with ketamine resulted in better sedation and muscle relaxation than dexmedetomidine alone [50]. Additionally, it has been reported that the anaesthetic effects of a combination of ketamine (5 mg/kg) with dexmedetomidine (10 μg/kg) were apparent within 3.2 ± 1.2 min [50]. In our study, the low dose of ketamine was probably the reason for the delayed time to maximum sedation compared to the study by Selmi et al. (2003) [50].

The combination of a_2_-agonists with opioids is often applied to achieve better sedative effects and a longer duration of action in veterinary patients [6]. In our study, in the DBT, DBP, and DT groups, sedation was significantly higher when compared to the control group (DC). Additionally, the administration of butorphanol significantly increased the sedative effect of dexmedetomidine, while the maximum sedation of the drug combination (T1–T2) was apparent within 13.3 ± 2.3 min. These results are in accordance with those reported in the literature. A dexmedetomidine–butorphanol combination caused apparent sedation very quickly (3.9 ± 2.1 min) [50], while the maximum sedative effect was reached within 10 min [20]. In the same manner, deeper sedation after the combination of dexmedetomidine and butorphanol in comparison to dexmedetomidine alone has been described [50]. Likewise, Slingsby et al. (2010) demonstrated deeper sedative results with the combination of dexmedetomidine and buprenorphine (20 and 10 μg/kg) than with either drug administered alone [8]. It has been reported that the administration of buprenorphine induces apparent sedation within a short time frame of 10–15 min, and the sedative effect reaches its peak within 45 min [57]. In our study, although the administration of buprenorphine increased sedation compared to the DC group, it did not significantly enhance dexmedetomidine sedation. However, a longer observation period may have altered the outcome. Similarly, a study in dogs by Cardoso et al. (2014) suggested that sedation levels were significantly higher after IM administration of a dexmedetomidine–tramadol combination in comparison to dexmedetomidine administered alone [58]. A study in cats by Hommuang et al. (2023) suggested that a combination of dexmedetomidine (20 μg/kg) and tramadol (1 mg/kg) administered intranasally induced an adequate sedative effect within 20 min [59]. However, there is insufficient information in the current literature comparing the IM administration of dexmedetomidine alone or in combination with tramadol in cats. In the present study, the administration of tramadol promptly increased dexmedetomidine sedation; however, this increase was not statistically significant. Nevertheless, the administration of a larger dose of tramadol could produce different results. In a study by Pypendop et al. (2009), the oral administration of 3–4 mg/kg of tramadol in cats produced sedative results, while a lower dose (0.5–1 mg/kg) produced euphoric behaviour [60]. In the same manner, the subcutaneous administration of tramadol (1 mg/kg) induced euphoric or dysphoric behaviour, although sedative results were not observed [61].

The mean duration of T1–T2 (10 min) was significantly shorter in the DT group in comparison to the DBT (*p* < 0.001) and DBP (*p* < 0.001) groups, suggesting that administration of tramadol may increase the level of sedation faster than butorphanol or buprenorphine. However, the sedation level was not significantly increased with the dose used. In our study, the administration of butorphanol significantly increased the sedative effect of dexmedetomidine, but the same result was not observed after the administration of buprenorphine. A longer duration of observation in our study may have produced different results because the IM administration of buprenorphine has a delayed onset and longer duration of action, while butorphanol has a rapid onset and short duration of action [13]. Nevertheless, in accordance with our results, it has been reported that the combination of dexmedetomidine (10 μg/kg) with butorphanol (0.4 mg/kg) produced a superior sedative effect in comparison to the combination of dexmedetomidine with buprenorphine (20 μg/kg) in cats [20].

### 4.2. Quality and Duration of Recovery

Atipamezole is a selective α_2_-adrenergic receptor antagonist that is administered to reverse the actions of medetomidine and dexmedetomidine [3,62,63,64]. The administration of atipamezole has also been reported to attenuate the effects of butorphanol in rats in a study by Jang and Lee (2009) [65]. Further research could be conducted to investigate the effects of atipamezole administration on other animal species, such as cats.

In our study, after the administration of atipamezole, the first signs of recovery appeared within 5 min, whereas recovery was completed within 20 min in all groups. These results are in accordance with those in the literature where, in various studies, the administration of atipamezole as an antagonist of a_2_-agonists in cats facilitated a calm, quick, and uneventful recovery that was observed within 6 [62], 10 [66], or 15 min [3]. The recovery assessment scales did not show a significant difference in recovery quality between the groups. However, the recovery scale scores were higher (worst recovery quality) in the DM group than in other groups. It is possible that a larger sample size or a higher dose of midazolam may have altered the outcome, and a significant difference could be detected. Specifically, in the DM group, 4/6 cats had an eventful recovery, exhibiting symptoms such as excitement, ataxia, trembling, and vocalisation. None of the cats in the other groups exhibited any similar symptoms. According to the literature, the primary metabolite of midazolam is 1-hydroxymidazolam, which is generally cleared and eliminated at a slower rate in cats than in dogs. The slow elimination rate of midazolam and its metabolites resulted in prolonged recovery in the two cats, as reported by Dholakia et al. (2019) [55]. Similarly, in our study, the time to full recovery (T3) after the administration of atipamezole (T2) was the most prolonged in the DM group (19.2 ± 1.9 min). This is most likely attributed to the excitement-like symptoms of midazolam, as described previously. The remaining cats in all groups recovered uneventfully with minimal ataxia. As mentioned above, various studies have described excitement-like symptoms of midazolam [19,40,52,55], which were also apparent throughout the recovery period of our study.

In the DK group, atipamezole was administered 30 min after the administration of ketamine (T2_A_), as suggested by the manufacturer, to avoid adverse effects. Additionally, the mean time to full recovery after the administration of atipamezole was 17.3 (±2.7) min, while all cats in group DK had a quite smooth recovery with minimal ataxia. It has been reported that, after the combination of ketamine with an a2-agonist and the reversal with atipamezole, convulsive activity, dysphoria, and oversensitivity may be displayed due to ketamine [63,67,68]. These results were not apparent in the cats in our study, possibly due to the low dose of ketamine used as well as the delayed administration of atipamezole.

### 4.3. Limitations

This study has several limitations. The small sample size may have led to an underestimation of the significant differences between the treatment groups. Subjective behavioural assessment scales have well-recognised inherent weaknesses. Regardless, the efficacy of the scale used is well established, as it has been used in multiple animal studies. Similarly, the scale was able to assess sedation and recovery quality based on clinical criteria. The fact that all cats had similar temperaments, had become accustomed to the researchers, and displayed no aggressive behaviour during the study lends support to the observed variations in sedation scores. Finally, two recovery assessment scales were used to minimise any potential bias, and most of the results were similar.

The results of our study correspond to well-behaved, young, and healthy cats. The drug combinations used may not produce adequate sedation in aggressive cats because the administered doses are relatively low. Nonetheless, the application of our drug combinations and the doses administered should be further investigated to be used in a clinical setting and in cats with different temperaments.

Another limitation was the necessity to interact with the cat every five minutes, which could have potentially aroused the patient. However, this was deemed necessary to increase the chances of identifying the time of maximum sedation, and all necessary handling and assessments were made using gentle, nonintrusive, and nonstressful techniques.

## 5. Conclusions

The results of our study suggest that insufficient sedation with dexmedetomidine may be enhanced by a second dose. Insufficient sedation with dexmedetomidine is also significantly improved by the addition of butorphanol and ketamine. Therefore, after an insufficient dose of dexmedetomidine, the subsequent administration of the appropriate dose of dexmedetomidine, butorphanol, or ketamine is recommended to enhance sedation in adult, healthy, well-behaved cats, while recovery after atipamezole administration is rapid and uneventful. On the other hand, the administration of midazolam at a low dose of 0.1 mg/kg has an adverse impact on dexmedetomidine sedation, resulting in a decreased sedation level and a challenging and protracted recovery period. Therefore, the administration of midazolam following an insufficient dose of dexmedetomidine is not recommended to increase the initial sedation in healthy adult cats.

## Figures and Tables

**Table 1:** Timeline of the experiments.

Time Point	Measurements and Administrations
T0	Sedation score evaluation (Grint) Administration of dexmedetomidine
q 5 min	Sedation score evaluation
T1	Sedation score evaluation (Grint) Administration of second drug
q 5 min	Sedation score evaluation (Grint)
T2	Sedation score evaluation (Grint) Administration of atipamezole
q 5 min	Recovery quality evaluation (Sams and Lozano)
T3	Full recovery

**Table 2:** The mean (±SD) duration (min) of all time intervals of the experiment is presented for seven groups of six adult cats that received different drug combinations.

Group	T0–T1	T1–T2	T0–T2	T2–T3	T0–T3
DD	19 (±1.9)	11.7 (±2.3)	30.8 (±3.4)	15.3 (±2.1)	46.2 (±4.7)
DC	18 (±2.5)	11.7 (±2.3)	29.2 (±1.9)	13.2 (±3.5)	42.3 (±4.95)
DT	15 (±0)	10 (±0)	25 (±0)	11.8 (±2.3)	36.8 (±2.3)
DBT	15 (±4.1)	13.3 (±2.3)	28.3 (±5.5)	11.3 (±1.97)	39.7 (±6.5)
DBP	16 (±1.9)	15 (±2.9)	30.8 (±1.9)	11.7 (±2.5)	42.5 (±2.6)
DK	18 (±2.5)	12.5 (±2.5)	29.2 (±1.9)	17.3 (±2.7)	46.5 (±4.03)
DM	17 (±3.7)	8.33 (±2.3)	25 (±4.1)	19.2 (±1.9)	44.2 (±4.5)
All	17 (±3.05)	11.8 (±3.05)	28.3 (±3.9)	14.3 (±3.8)	42.6 (±5.5)

T0: baseline, dexmedetomidine administration. T1: maximum sedation with dexmedetomidine–second drug administration. T2: maximum sedation with the drug combination. T3: time point of full recovery. Group DD: administration of two subsequent doses of dexmedetomidine; DC: dexmedetomidine–saline combination (control group); DT: dexmedetomidine–tramadol combination; DBT: dexmedetomidine–butorphanol combination; DBP: dexmedetomidine–buprenorphine combination; DK: dexmedetomidine–ketamine combination; DM: dexmedetomidine–midazolam combination.

**Table 3:** Comparison of the mean duration of T1–T2 and T0–T2 between the seven groups of six adult cats that received different drug combinations.

Mean duration of T1–T2	**Group**	**DD** (*p* value)	**DC** (*p* value)	**DT** (*p* value)	**DBT** (*p* value)	**DBP** (*p* value)	**DK** (*p* value)
	DC	(0.967)					
	DT	TR = 0.781 (0.002)	TR = 0.784 (0.021)				
	DBT	(0.308)	(0.314)	TR = 1.41 (<0.001)			
	DBP	TR = 1.263 (0.014)	TR = 1.268 (0.011)	TR = 1.617 (<0.001)	(0.160)		
	DK	(0.548)	(0.563)	TR = 1.363 (0.001)	(0.723)	(0.118)	
	DM	TR = 0.727 (0.001)	TR = 0.73 (0.001)	(0.476)	TR = 0.66 (<0.001)	TR = 0.57 (<0.001)	TR = 0.68 (<0.001)
Mean duration of T0–T2	**Group**	**DD** (*p* value)	**DC** (*p* value)	**DT** (*p* value)	**DBT** (*p* value)	**DBP** (*p* value)	**DK** (*p* value)
	DC	(0.431)					
	DT	TR = 0.812 (<0.001)	TR = 0.86 (0.026)				
	DBT	(0.224)	(0.496)	(0.124)			
	DBP	(0.924)	(0.412)	TR = 1.23 (0.002)	(0.134)		
	DK	(0.661)	(0.707)	TR = 1.194 (0.009)	(0.291)	(0.657)	
	DM	TR = 0.808 (0.004)	TR = 0.847 (0.016)	(0.84)	(0.082)	TR = 0.8 (<0.001)	TR = 0.826 (0.005)

All confidence intervals were set at 95%. If the difference is statistically significant, then the time ratio (TR) value is also presented. A TR > 1 suggests that the duration was significantly prolonged for the group in the row compared to the group in the column. A TR < 1 suggests that the duration was significantly shorter for the group in the row than for the group in the column. The *p* values are also presented in parentheses. T0: baseline, dexmedetomidine administration. T1: maximum sedation with dexmedetomidine and administration of the second drug. T2: maximum sedation with the drug combination. Group DD: administration of two repeated doses of dexmedetomidine; DC: dexmedetomidine–normal saline 0.9% combination (control group); DT: dexmedetomidine–tramadol combination; DBT: dexmedetomidine–butorphanol combination; DBP: dexmedetomidine–buprenorphine combination; DK: dexmedetomidine–ketamine combination; DM: dexmedetomidine–midazolam combination.

**Table 4:** Median (range) scores for the Grint sedation scale at time points T1 and T2 for seven groups of six adult cats that received different drug combinations.

Group	Grint Score	
	T1	T2
DD	7.5 (6–9)	10 (8–12)
DC	6 (5–7)	5 (4–6)
DT	7 (6–9)	8 (7–11)
DBT	6.5 (6–8)	9 (7–12)
DBP	6.5 (5–8)	10 (5–11)
DK	6 (5–7)	10 (8–11)
DM	7 (6–8)	7.5 (3–9)

At T0 (baseline), according to Grint sedation scale scores, the baseline score was zero in all groups. T1: maximum sedation with dexmedetomidine–second drug administration. T2: maximum sedation with the drug combination. Group DD: administration of two repeated doses of dexmedetomidine; DC: dexmedetomidine–NS 0.9% combination (control group); DT: dexmedetomidine–tramadol combination; DBT: dexmedetomidine–butorphanol combination; DBP: dexmedetomidine–buprenorphine combination; DK: dexmedetomidine–ketamine combination; DM: dexmedetomidine–midazolam combination.

**Table 5:** Comparison of the median Grint sedation scale score differences at intervals T1–T2 and T0–T2 between seven groups of six adult cats that received different drug combinations.

Grint scale score differences at interval T1–T2	**Group**	**DD** (*p* value)	**DC** (*p* value)	**DT** (*p* value)	**DBT** (*p* value)	**DBP** (*p* value)	**DK** (*p* value)
	DC	Coef.: −3 (0.006)					
	DT	Coef.: −2 (0.043)	Coef.: 3 (0.033)				
	DBT	(0.297)	Coef.: 4 (0.001)	(0.107)			
	DBP	(0.297)	Coef.: 4 (0.004)	(0.107)	(1.000)		
	DK	Coef.: 2 (0.049)	Coef.: 4 (0.001)	Coef.: 2 (0.002)	(0.174)	(0.414)	
	DM	(0.328)	(0.328)	(0.372)	(0.353)	(0.170)	(0.128)
Grint scale score differences at interval T0–T2	**Group**	**DD** (*p* value)	**DC** (*p* value)	**DT** (*p* value)	**DBT** (*p* value)	**DBP** (*p* value)	**DK** (*p* value)
	DC	Coef.: −5 (0.001)					
	DT	Coef.: −2 (0.008)	Coef.: 3 (0.012)				
	DBT	(0.203)	Coef.: 4 (0.001)	(0.321)			
	DBP	(1.000)	Coef.: 5 (0.001)	(0.069)	(0.170)		
	DK	(1.000)	Coef.: 5 (0.001)	Coef.: 2 (0.003)	(0.170)	(1.000)	
	DM	(0.346)	(0.814)	(0.607)	(0.506)	(0.335)	(0.335)

If the difference is statistically significant, then the co–efficiency (Coef.) value is also presented. A Coef. > 0 indicates that the sedation score was significantly higher in the row group than in the column group. A Coef. < 0 indicates that the sedation score was significantly lower for the group in the row than for the group in the column. All confidence intervals were set at 95%. The *p* values are presented in parentheses. T1: maximum sedation with dexmedetomidine and administration of the second drug. T2: maximum sedation with the drug combination. Group DD: administration of two repeated doses of dexmedetomidine; DC: dexmedetomidine–NS 0.9% combination (control group); DT: dexmedetomidine–tramadol combination; DBT: dexmedetomidine–butorphanol combination; DBP: dexmedetomidine–buprenorphine combination; DK: dexmedetomidine–ketamine combination; DM: dexmedetomidine–midazolam combination.

**Table 6:** Comparison of the mean duration of T2–T3 (T2_A_–T3 for the DK group) between seven groups of six adult cats that received different drug combinations.

Group	DD	DC	DT	DBT	DBP	DK
DC	(0.468)					
DT	TR = 0.79 (0.039)	(0.150)				
DBT	TR = 0.755 (0.003)	TR = 0.81 (0.025)	(0.686)			
DBP	TR = 0.787 (0.011)	(0.071)	(0.973)	(0.662)		
DK	(0.247)	(0.071)	TR = 1.42 (<0.001)	TR = 1.49 (<0.001)	TR = 1.43 (0.001)	
DM	(0.061)	TR = 1.29 (0.011)	TR = 1.52 (<0.001)	TR = 1.59 (<0.001)	TR = 1.53 (<0.001)	(0.649)

All confidence intervals (CI) were set at 95%. If the difference is statistically significant, then the time ratio (TR) value is presented. A TR > 1 suggests that the duration was significantly prolonged for the group in the row compared to the group in the column. A TR < 1 suggests that the duration was significantly shorter for the group in the row than for the group in the column. The *p* values are also presented in parentheses. T2: maximum sedation with the drug combination. T2_A_ time of administration of atipamezole in the DK group. T3: time point of full recovery. Group DD: administration of two repeated doses of dexmedetomidine; DC: dexmedetomidine–NS 0.9% combination (control group); DT: dexmedetomidine–tramadol combination; DBT: dexmedetomidine–butorphanol combination; DBP: dexmedetomidine–buprenorphine combination; DK: dexmedetomidine–ketamine combination; DM: dexmedetomidine–midazolam combination.

## Data Availability

Applicable upon request.

## Supplementary Materials

The following supporting information can be downloaded at: https://www.mdpi.com/article/10.3390/vetsci11050186/s1, Table S1: The composite descriptive sedation scores by Grint et al.; Table S2: The Simple Descriptive Scale (SDS) developed by Lozano et al.; Table S3: Ataxia, induction, and recovery quality scores reported by Sams et al.; Table S4: Comparison of the median Grint sedation scale scores between T1 and T2 and between T0 and T2 in seven groups of adult cats that received different drug combinations.
