# Supplementary material for: The Effect of a Subsequent Dose of Dexmedetomidine or Other Sedatives following an Initial Dose of Dexmedetomidine on Sedation and Quality of Recovery in Cats: Part I
Source: Vet Sci. 2024 Apr 25;11(5):186. doi: 10.3390/vetsci11050186 (PMC11126131; doi:10.3390/vetsci11050186)
Supplement: Supplementary file 1 [file vetsci-11-00186-s001.zip › vetsci-2877023-supplementary.pdf]

Sedation quality assessment scale:

Table S1: The composite descriptive sedation scores by Grint et al.

The scores of this scale range from 0 to 21, with zero indicating no sedation and 21 indicating deep sedation. Seven attributes were examined, namely the spontaneous posture (score 0-4), the palpebral reflex (score 0-3), the position of the eyes (score 0-2), the relaxation of the jaw and tongue (scores 0-3), the response to a standardized noise (handclap) (scores 0-3), the resistance when the animal was laid laterally (scores 0-3), and the general appearance of the animal (scores 0-3). The spontaneous posture of the animal could be described as: standing (0), tired but standing (1), lying but able to rise (2), lying but able to rise with difficulty (3), or unable to rise (4). The palpebral reflex could be described as: brisk (0), slow with full appearance of the cornea (1), slow with partial appearance of the cornea (2), or absent (3). The eye position could be characterised as: central (0), rotated downwards but not obscured by the third eyelid (1), or rotated downwards and obscured by the third eyelid (2). The jaw tone and tongue relaxation could be characterised as: normal (0), reduced (1), moderately reduced (2), or complete loss of tone (3). The response of the animal to noise can be described as: normal (0), reduced (1), minimal (2), or absent (3). The animal's resistance or struggle when laid into lateral recumbency can be described as: intense (0), moderate (1), minimal (2), or no struggling (3). Finaly, the general appearance of the animal could be characterised as: excitable (0), awake (1), calm (2), or apathetic (3).

Recovery quality assessment scales:

Table S2. The Simple Descriptive Scale (SDS) developed by Lozano et al.

The scores on this scale ranged from one to five. A score of one indicated a very smooth recovery, with no adverse reactions. A score of two indicated a quite smooth recovery, with little excitement. A score of three indicated a moderately smooth recovery, with excitement and mild adverse reactions. A score of four indicated a recovery that was not smooth, with excitement and adverse reactions, and finally a score of five indicated an extremely violent recovery, with need for anticonvulsant therapy.

Table S3. Ataxia, induction, and recovery quality scores reported by Sams et al.

The scores on this scale varied from zero to three. The score of zero indicated a perfect and smooth recovery, without ataxia; the score of one indicated a good and uncomplicated recovery, with minimal ataxia present; the score of two indicated a difficult recovery with moderate ataxia present and the score of three indicated a rough recovery with significant ataxia present.

Table S4. Comparison of the median Grint sedation scale scores between T1 and T2 and between T0 and T2 in seven groups of adult cats that received different drug combinations.

GROUP	**T1-T2** (*p* value)	**T0-T2** (*p* value)
DD	0.0313	0.0313
DC	0.0625	0.0313
DT	0.125	0.0313
DBT	0.0313	0.0313
DBP	0.0625	0.0313
DK	0.0313	0.0313
DM	0.562	0.0313

The *p* values are presented in the parentheses. T0: baseline, dexmedetomidine administration; T1: maximum sedation with dexmedetomidine–2nd drug administration; T2: maximum sedation with the drug combination and administration of atipamezole. Group DD: administration of two repeated doses of dexmedetomidine; DC: dexmedetomidine–NS 0.9% combination (control group); DT: dexmedetomidine–tramadol combination;

DBT: dexmedetomidine–butorphanol combination; DBP: dexmedetomidine–buprenorphine combination; DK: dexmedetomidine–ketamine combination; DM: dexmedetomidine–midazolam combination.
